# Involvement of Cdk5 activating subunit p35 in synaptic plasticity in excitatory and inhibitory neurons

**DOI:** 10.1186/s13041-022-00922-x

**Published:** 2022-04-28

**Authors:** Miyuki Takahashi, Takeru Nakabayashi, Naoki Mita, Xiaohua Jin, Yuta Aikawa, Kodai Sasamoto, Goichi Miyoshi, Mariko Miyata, Takafumi Inoue, Toshio Ohshima

**Affiliations:** 1 Laboratory for Molecular Brain Science, Department of Life Science and Medical Bioscience, Waseda University, 2-2 Wakamatsu-cho, Shinjuku, Tokyo 162-0056 Japan; 2 Laboratory for Neurophysiology, Department of Life Science and Medical Bioscience, Waseda University, 2-2 Wakamatsu-cho, Shinjuku, Tokyo 162-0056 Japan; 3 Department of Neurophysiology, Tokyo Women’s Medical University, 8-1 Kawada-cho, Shinjuku, Tokyo 162-8666 Japan; 4 Department of Genetic and Behavioral Neuroscience, Gunma University Graduate School of Medicine, 3-39-22 Showa-cho, Maebashi, Gunma 371-8511 Japan

**Keywords:** Synaptic plasticity, Learning and memory, Long-term depression, Long-term potentiation

## Abstract

Cyclin-dependent kinase 5 (Cdk5) /p35 is involved in many developmental processes of the central nervous system. Cdk5/p35 is also implicated in synaptic plasticity, learning and memory. Several lines of conditional Cdk5 knockout mice (KO) have been generated and have shown different outcomes for learning and memory. Here, we present our analysis of p35 conditional KO mice (p35cKO) in hippocampal pyramidal neurons or forebrain GABAergic neurons using electrophysiological and behavioral methods. In the fear conditioning task, CamKII-p35cKO mice showed impaired memory retention. Furthermore, NMDAR-dependent long-term depression (LTD) induction by low-frequency stimuli in hippocampal slices from CamkII-p35cKO mice was impaired compared to that in control mice. In contrast, Dlx-p35cKO mice showed no abnormalities in behavioral tasks and electrophysiological analysis in their hippocampal slices. These results indicated that Cdk5/p35 in excitatory neurons is important for the hippocampal synaptic plasticity and associative memory retention.

**Supplementary Information:**

The online version contains supplementary material available at 10.1186/s13041-022-00922-x.

## Introduction

Cyclin-dependent kinase 5 (Cdk5) is a proline-directed serine/threonine kinase that belongs to the CDK family and is expressed primarily in the central nervous system. Cdk5 plays a critical role in brain development, neuronal migration and survival, and regulates multiple aspects of dendrite development, synaptic plasticity, learning and memory [1–4]. Cdk5 is activated by complexing with neuron-specific activator molecules such as p35 and p39. The association between Cdk5 and its activators is essential for the kinase activation [5–7]. Cdk5 knockout mice display perinatal lethality [8], whereas p35 and p39 null mice are viable. Mice lacking p35 show lamination defects in the cerebral cortex but experience only mild disruptions in the hippocampus and have fairly normal cerebella [9]. On the contrary, p39 deficient mice do not exhibit detectable abnormalities in neuronal positioning. However, the phenotypes of p35; p39 double-knockout mice and Cdk5 null mutant mice are identical, which strongly suggests that p35 and p39 are the only activators of Cdk5 [10]. In our previous study, we generated and analyzed CreER-p35 conditional knockout mice (cKO), in which p35 is inducibly deleted in the brain. CreER-p35cKO mice show reduced dendritic spine density in CA1 pyramidal neurons and impaired LTD induction in the hippocampus with impairment in spatial learning and memory and reduced anxiety-like behavior [11]. Since these mice had p35 deletion in all cells, it was impossible to separate and analyze its functions in excitatory and inhibitory neurons.

In the present study, we created mice in which the p35 gene was deleted in hippocampal excitatory neurons (CaMKII-Cre p35cKO) or GABAergic inhibitory neurons (Dlx-Cre p35cKO). Using behavioral and electrophysiological analyses, we investigate whether the p35/Cdk5 activity is involved in associative memory learning in excitatory or inhibitory neurons.

## Materials and methods

### Animal experiments

All experimental protocols were approved by the Institutional Animal Care and Use Committee of Waseda University. Throughout the experiment, efforts were made to minimize the number of animals used and their suffering. Mice were fed ad libitum with standard laboratory food and water in animal cages under a 12 h light/dark cycle. *p35*-flox mice were generated on a C57BL/6 J background [12]. CaMKII-Cre mice [13] were obtained from the Jackson Laboratory (stock number 005359). CaMKII-Cre (CA1-p35 cKO) mice were obtained by crossing p35 flox/ + ; CaMKII-Cre and p35 flox/flox mice. Cre activity is expressed in the hippocampal CA1 region after P17 [13, 14]. Dlx5/6-Cre (Dlx-Cre) mice [15] were obtained from the Jackson Laboratory (stock number 008199). Dlx-Cre expresses Cre recombinase in GABAergic neurons in the forebrain from E13.5 [15, 16]. The genotype of these mice was determined by PCR using DNA obtained from tail biopsies as described previously [12].

### Mouse behavior experiments

Behavioral tests were conducted with adult male mice of 8–25 weeks of age, using either p35f/f mice and CamkII-Cre p35cKO or Dlx-Cre p35cKO littermates, according to previously described methods. Behavioral experiments were carried out during the light phase on the same day after the mice were acclimated to a test room 1 h prior to testing. Mouse behavior was recorded using a video camera and analyzed with each software described as below (O’Hara &Co., Japan).

### Open field

Spontaneous activities of mice were evaluated in an open field (60 cm × 60 cm) at 80 lx for 60 min. The mice were placed in the center of the open field, and their locomotive activity was recorded and analyzed using the OFT software (O’Hara &Co., Japan).

### Novel object recognition test

Mice were allowed to explore two identical objects in a test box for 10 min, and then returned to their home cages. After 1 h, they were returned to the test box, in which familiar and novel objects were placed, and were allowed to explore and investigate the objects. The time spent on familiar or novel objects was monitored with a video camera and the images were processed using the OFT software (O’Hara &Co., Japan).

### Contextual fear-conditioning test

A fear-conditioned shock chamber (17 cm × 10 cm × 10 cm) was used. Mice were placed in the conditioning chamber and allowed to explore for 1 min. An electric foot shock (0.7 mA, 2 s) was given during the last of a tone, and this session was repeated three times at intervals of 10 s. After 24 h, a contextual test was performed. The mice were returned to the same test chamber and their freezing time was monitored for 3 min without shock and cues. 24 h later, the cued test was performed. Mice were returned to another test chamber, and their freezing time was monitored for 3 min with only a tone. The freezing time of the mice was monitored using a video camera, and images were processed using NIH Image FZ software (O’Hara &Co., Japan).

### Passive avoidance test

The passive avoidance (PA) task was conducted according to a previously described method [17, 18] using a step-through PA apparatus (MPB-M001; Melquest, Japan). It consists of a large white-painted illuminated compartment (26 × 26 × 34 cm) and a small black-painted dark compartment (13 × 7.5 × 7.5 cm) separated from each other by a guillotine gate. For the acquisition trial, each mouse was placed in the illuminated compartment for 30 s, and the gate was opened. As soon as the mouse entered the dark compartment, the gate was closed and an electrical shock (0.25 mA, 3 s) was delivered through the grid floor using a shock generator (SG-100, Melquest, Japan). For the retention trial, the mice were placed in the illuminated white compartment and the latency time between door opening and entry into the dark compartment was recorded for each mouse. The cutoff latency was set to 180 s. The retention trial was conducted 1 d after the acquisition trial.

### Electrophysiological analysis

Acute hippocampal slices were prepared according to a standard procedure [19] with a slice-cutting solution containing (mM): 120 choline Cl, 3 KCl, 8 MgCl_2_, 1.25 NaH_2_PO_4_, 26 NaHCO_3_, and 20 glucose, kept at 0 °C during cutting. Artificial cerebrospinal fluid (ACSF, in mM): 124 NaCl, 2.5 KCl, 2 CaCl_2_, 2 MgCl_2_, 1.25 NaH_2_PO_4_, 26 NaHCO_3_, and 20 glucose, bubbled continuously with a mixture of 95% O_2_ and 5% CO_2_, was used for incubation and recording at room temperature (23–25 °C). Slices were cut at a thickness of 400 μm using a vibratome-type tissue slicer (Pro7; Dosaka EM, Kyoto, Japan). A bipolar stimulation electrode was placed in the Schaffer collateral, and a glass micropipette filled with ACSF (3–6 MΩ) was placed in the stratum radiatum of the CA1 region to record field excitatory postsynaptic potentials (fEPSPs) with an amplifier (M-707, World Precision Instruments, Sarasata, FL, U.S.A.). In the LTD and LTP studies, the test stimulation was delivered every ten seconds. If the average in any 2 min period during the 20 min base line period just before LTD or LTP induction stimuli exceeded ± 5% of the baseline average, the records were discarded. Electrophysiological data were fed to a Mac computer running in-house software, TI WorkBench [20], through an interface (NI USB-6211, National Instruments, Austin, TX, U.S.A.) at a 20 kHz sampling rate after low-pass filtering at 0.5 kHz with a 4 Pole Bessel filter (LPF-202A, Warner Instruments, Hamden, CT, U.S.A.). Statistical analysis was conducted by two-way repeated measures ANOVA, and the mean ± SEM is shown in the graph. Statistical significance was set at p < 0.05. In experiments using p35f/f and Dlx-p35cKO mice, LTP experiments were performed using 4- to 8-month-old male mice. LTP was induced by theta burst stimulation (TBS). TBS was composed of 100 Hz × 4 stimuli repeated ten times at 5 Hz, which was applied four times at 10 s interval. LTD experiments were performed on postnatal day (P) 10–14 old male mice. LTD was induced by 1 Hz stimulation for 900 s. In experiments on CamKII-p35cKO and wild-type (WT) mice as control, 16- to 20-week-old mice were used in LTP experiments and P 28–35 mice were used in LTD experiments, where LTP was induced by tetanic stimulation (100 Hz for 1 s) and LTD was induced by combining 1 Hz, 900 s stimulation with a glutamate transporter inhibitor *L*-trans-pyrrolidine-*2*,*4*-dicarboxylic acid (tPDC) [21].

## Results

We previously reported phenotypic analysis of Dlx-p35cKO;p39KO mice [22]. In this double-knockout mouse, we confirmed a reduction of p35 protein in the striatum, which consists mostly of GABAergic neurons [22]. In CaMKII-p35cKO hippocampus, reduction of p35 protein was confirmed in our previous study [14]. In the present study, we created mice in which the p35 gene was deleted only in hippocampal excitatory neurons (CaMKII-Cre p35cKO) or GABAergic inhibitory neurons (Dlx-Cre p35cKO) to investigate whether the p35/Cdk5 activities in excitatory or inhibitory neurons are involved in learning and memory.

### Behavioral analyses

**Fig. 1:**
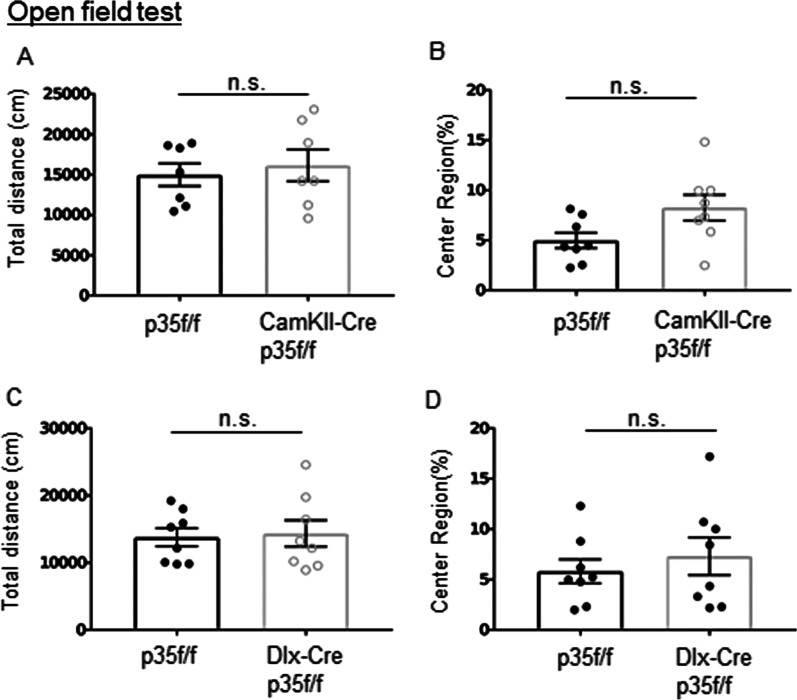
Open field test. **A**, **C** The total distance in the open field arena was measured for 60 min of free movement. **B**, **D** Percentage of entries in the center of the open field during activity measurement time (means ± SEM, n = 8 for p35f/f, CamkII-p35cKO and Dlx-p35cKO mice, ns, not significant, Mann–Whitney U-test)

We first observed the locomotive activity of CamkII-p35cKO and Dlx-p35cKO mice using an open-field test. No significant difference was detected in CamkII-p35cKO mice in total distance compared to p35f/f mice (Fig. 1A), and the ratio of entering the center of the open field arena was also similar (Fig. 1B). We then examined the locomotive movement of Dlx-p35cKO mice compared with that of p35f/f mice. Similar to CamKII-p35cKO mice, no significant difference was found (Fig. 1C). In addition, no significant difference was detected between Dlx-p35cKO mice and p35f/f mice in relation to the spent time at center of the field (Fig. 1D). Indicating that p35 deficiency in either excitatory or inhibitory neurons had no effect on the locomotive activity.

When an object was placed in one corner of the open field arena, CamkII-p35cKO and p35f/f mice spent the same amount of time on the object (Additional file 1: Fig. S1A). We wondered whether the lack of p35 affects cognitive ability. To investigate this possibility, we placed another novel object and measured the time spent interacting with the familiar or novel object. CamkII-p35cKO mice spent longer time on the novel object than on the familial object, and the interaction time was similar to that of WT mice (Additional file 1: Fig. S1B). Similarly, for Dlx-p35cKO mice, when the novel object was placed in one corner of the open field arena, Dlx-p35cKO and p35f/f mice spent the same amount of time on the object (Additional file 1: Fig. S1C). Furthermore, we performed the same experiment on Dlx-p35cKO mice: while Dlx-p35cKO mice also spent longer time on the novel object than the familial one, the duration was similar to that of WT mice (Additional file 1: Fig. S1D), suggesting that p35 deficiency did not affect object recognition ability.

We also performed Y-maze test, which is used to confirm memory function. WT mice remember entering one of the three arms and tend to avoid the arm they have already entered. This habit was used to test the memory function. The number of entries into the three arms was not significantly different between CamKII-p35cKO and p35f/f mice (Additional file 2: Fig. S2A), and the alternation rate was also not different (Additional file 2: Fig. S2B). Similarly, in the case of Dlx-p35cKO, the number of arms entered was not significantly different (Additional file 2: Fig. S2C), and the alternation rate was also not different (Additional file 2: Fig. S2D).

**Fig. 2:**
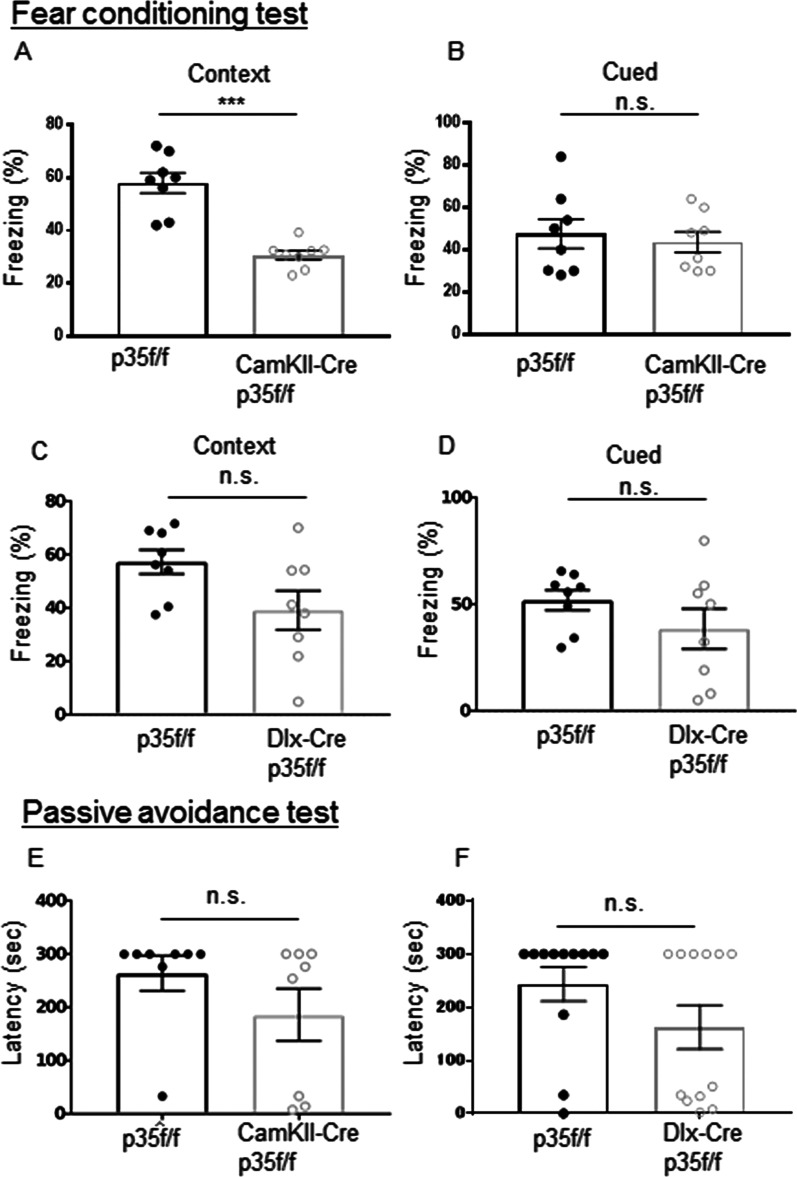
Fear conditioning test. **A**–**D** Contextual fear conditioning test. Freezing time during trials are shown. **A**, **C** Freezing response in the same chamber as contextual fear conditioning (means ± SEM, n = 8 for p35f/f, CamkII-p35cKO and Dlx- p35cKO mice, ***p < 0.0001, Mann–Whitney U-test). **B**, **D** Freezing response with cue test (means ± SEM, n = 8 for p35f/f, CamkII-p35cKO and Dlx- p35cKO mice, ns, not significant, Mann–Whitney U-test). **E**, **F** Passive avoidance test. Latency time between door opening and entry into the dark compartment (means ± SEM, n = 8 for p35f/f, CamkII-p35cKO and Dlx- p35cKO mice, ns, not significant, Mann–Whitney U-test)

Subsequently, we conducted a fear-conditioning test. Twenty four h after the conditioning test, CamkII-p35cKO mice showed significantly reduced freezing behaviors when re-exposed to the shock-paired context (Fig. 2A), but not after presentation of the tone cue in an altered context (Fig. 2B). In contrast, Dlx-p35cKO mice showed no significant difference from p35-flox mice in context (Fig. 2C) and cued (Fig. 2D) tests. Together, these findings suggest that the loss of p35 in excitatory pyramidal neurons causes significant deficits in fear learning and memory. We then conducted a passive avoidance test to examine hippocampus-dependent memory in both the p35cKO lines. During the training session, control and Dlx-p35cKO or CamkII-p35cKO mice were placed in the light compartment and showed a similar latency in entering the dark compartment, where they received a foot shock. In the test session, 24 h after training, the mice were placed in the lighted compartment again. The CamkII-p35cKO (Fig. 2E) and Dlx-p35cKO (Fig. 2F) mice showed comparable latency in entering the dark compartment to the p35f/f mice.

### Electrophysiological analyses

In the fear conditioning test, CamkII-p35cKO mice showed significantly reduced freezing behavior when re-exposed to the shock-paired context (Fig. 2A), suggesting that p35 deficiency in the CamkII expression region has a significant effect on synaptic plasticity.

**Fig. 3:**
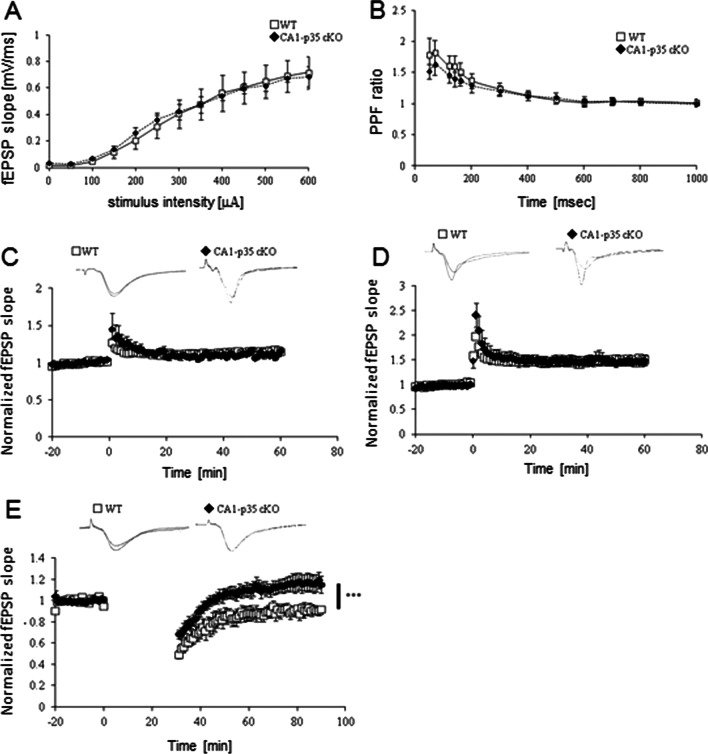
Altered synaptic plasticity in hippocampal CA1 in CA1-p35 cKO mice. **A** I–O curve of the Shaffer collateral-CA1synapse in CA1-p35 cKO and WT mice. There was no clear difference in synaptic strength between the two genotypes (n = 5). **B** Paired-pulse facilitation (PPF) ratio was not altered in CreER-p35 cKO mice compared with that in p35-flox mice (n = 5). **C** LTP-induction by TBS in hippocampal CA1 in CA1-p35 cKO was similar to that in WT mice (n = 5). **D** LTP-induction by tetanus stimuli in hippocampal CA1 in CA1-p35 cKO mice were not distinguishable from that in WT mice (n = 6). **E** NMDAR-dependent LTD with glutamate transporter inhibition in hippocampal CA1 in CA1-p35 cKO and WT mice. NMDAR-dependent LTD was significantly impaired in CA1-p35 cKO mice (n = 5). Error bars represent SEM. ***, p < 0.001, two-way repeated-measures ANOVA

To further investigate the specific role of Cdk5/p35, a set of electrophysiological experiments at the Schaffer collateral-CA1 synapse was conducted to evaluate postsynaptic-specific loss of Cdk5/p35 function in synaptic plasticity (Fig. 3). The input–output (I-O) curves showed that the synaptic strength in CA1-p35cKO mice was similar to that in WT mice (Fig. 3A). No difference was found in the magnitude of paired pulse facilitation (PPF, Fig. 3B), indicating there was no obvious presynaptic dysfunction. No difference was observed in the magnitude of LTP by theta-burst stimulation (TBS) and tetanus stimuli between CA1-p35cKO and WT mice (Fig. 3C, D). Intriguingly, NMDAR-dependent LTD induction by low-frequency stimuli with the inhibition of glutamate transporter tPDC in CA1-p35cKO mice was impaired (Fig. 3E).

**Fig. 4:**
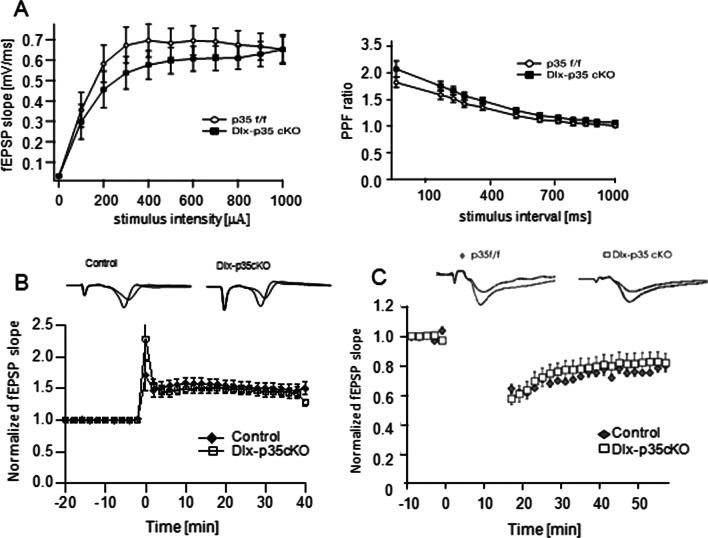
Synaptic functions at Shaffer collateral-CA1 pyramidal neuron synapse in Dlx-p35cKO mice. **A** I–O curve (left) and PPF ratio (right) of the Shaffer collateral-CA1synapse in Dlx-p35cKO and control p35 f/f mice. Synaptic strength was not apparently different, and PPF ratio was not altered in Dlx-p35cKO mice. **B** LTP induction by TBS was not altered in Dlx-p35 cKO mice. **C** LTD induction in hippocampal CA1 in P10-18 Dlx-p35 cKO mice resulted in no significant difference from that in p35 f/f mice (n = 5). Top traces indicate overlapped fEPSP wave forms just before and 40 min after LTP induction

We also compared the synaptic functions of Dlx-p35cKO mice and p35f/f mice as control at the Shaffer collateral-CA1 synapses in acute hippocampal slices. The I-O relationship and PPF were comparable (Fig. 4A). The magnitude of LTP induced by TBS in Dlx-p35cKO mice was not different (Fig. 4B). LTD induced by 1 Hz low-frequency stimulation was also not different between the control and Dlx-p35cKO mice (Fig. 4C). Taken together, these results indicate that postsynaptic Cdk5/p35 in the hippocampal CA1 plays a vital role in NMDAR-dependent LTD induction rather than LTP induction, while the lack of Cdk5/p35 in inhibitory interneurons do not have overt effect at the Shaffer collateral-CA1 excitatory synapses.

## Discussion

In our previous study using inducible p35cKO mice, we demonstrated the functional loss of p35 impaired spatial learning and memory [11]. In addition, we observed normal LTP induction but disturbed LTD induction in hippocampal slices [11]. In the present study, we conditionally inactivated p35 in excitatory neurons of the hippocampal CA1 (CamKII-Cre p35cKO) or inhibitory neurons (Dlx-Cre p35cKO) and subsequently conducted behavioral and electrophysiological analyses of hippocampal slices. In a previous study using the Cdk5 inhibitor butyrolactone I, Cdk5 activity was shown to be required for associative learning [23]. However, there are no reports of associative learning in mutant mice that lack or have reduced Cdk5 activity. Thus, in the present study, we examined associative learning using CamKII-p35cKO and Dlx-p35cKO mice. We observed impairments in associated memory in the fear condition task in CamKII-p35cKO mice but not in Dlx-p35cKO mice (Fig. 2A). We identified a comparative response in the cue test in CaMKII-Cre p35cKO mice (Fig. 2B). We used CaMKII-cre, in which cre is expressed in a CA-1 specific manner [13, 14]. To investigate the involvement of p35/Cdk5 in cued fear conditions, other types of cre mice should be used to delete the p35 gene in other regions of the brain, including the amygdala [24] in future studies. LTP induction in CA1 was normal in both p35cKO mouse lines, but LTD induction was impaired in CamKII-p35cKO mice (Fig. 3E), indicating that p35 in excitatory neurons is critical for LTD induction in hippocampal CA1 neurons and associated memory formation. In this study, we used only male mice for electrophysiological and behavioral studies. As p25 or p35 manipulations can have sexually dimorphic effects [4, 25], it would be interesting to also study female CamKII-p35cKO mice**.** We conducted LTP experiments using 4–8 month-old mice. Since LTP mechanisms switch with development [26], the study of LTP in younger p35cKO mice would also be useful to delineate the age-dependent role of p35/Cdk5 in LTP.

Recent studies involving Cdk5 cKO mice have reported contradictory results regarding synaptic plasticity. Inducible Cdk5 cKO mice showed enhanced synaptic plasticity [27], whereas CA1-specific Cdk5 cKO mice showed impaired synaptic plasticity [28]. Cell type-specific conditional KO was conducted using parvalbumin (PV)-Cre mice [29]. PV-Cdk5cKO mice showed impaired LTP induction, which was rescued by expression of picrotoxin in hippocampal CA1 [29].

In this study, we observed impaired associative memory formation (Fig. 2A) and defects in hippocampal LTD (Fig. 3E) in CamKII-p35cKO mice. In Bax CA1-cKO mice, associative memory retention 24 h after conditioned stimuli was impaired, and hippocampal LTD was defective [30]. A similar phenotype has been reported in downstream regulatory element antagonist modulator (DREAM) Tg mice [31]. However, the relationship between Cdk5/p35 and these molecules remains unclear. It is still not known why the loss of p35 causes impairment of hippocampal LTD [3, 14]. Ca^2+^-dependent activation of hippocalcin via Ca^2+^ influx is implicated in hippocampal LTD [32], leading to the formation of a complex with AP-2, which is part of the clathrin-mediated endocytic machinery. Cdk5 is involved in clathrin-mediated endocytosis via phosphorylation of amphiphysin I and dynamin I [33, 34]. NMDA-dependent p35 cleavage and subsequent p25/Cdk5 activation have been described and shown to mediate NMDA-dependent LTD in the hippocampus [35]. Deletion of the p35 gene results in a lack of p25 expression; our results of the hippocampal LTD study in CaMKII-p35cKO mice (Fig. 3E) are consistent with those of a previous study which found disturbed LTD induction in Δp35KI mice, in which p25 is not produced by eliminating the cleavage site of p35 by calpain [35]. Using hippocampal slices from Δp35KI mice, Seo et al. showed the involvement of p25/Cdk5 in AMPAR endocytosis via inhibition of DARPP-32 and activation of PP1 and calcineurin in response to neural activation [35]. Our results support these findings.

A deficit in hippocampal LTD has been reported in mice lacking PSD-95 [36], which is a major postsynaptic scaffold protein in glutamatergic synapses [37]. PSD-95 interacts with AMPARs by binding to stargazin [38–40]. Cdk5 phosphorylates PSD-95 and regulates its ubiquitination, which is implicated in AMPA receptor endocytosis during LTD [41]. In a previous study, p35 was shown to be involved in the extinction of contextual fear memories [42]. Thus, it would be interesting to study the extinction of contextual fear memory in CamKII-p35cKO mice.

In summary, the present study demonstrated a significant role for Cdk5/p35 in excitatory neurons in the mouse hippocampus in associative memory formation and hippocampal synaptic plasticity.

## Supplementary Information

**Additional file 1: Figure S1.** Novel object recognition (A, C) The mouse was placed in the open field in which the object was placed, and the time spent on the object was measured for 10 min. (B, D) After (A, C), a novel object was placed in the other corner and the time spent by the mouse on a familiar or novel object was measured for 10 min. (mean ± SEM, n = 8 for p35f/f, CamkII-p35cKO and Dlx- p35cKO mice,*p < 0.05 for object two-way repeated- measures ANOVA, ns, not significant).

**Additional file 2: Figure S2.** Y-maze test. (A, C) The number of times the mouse entered the three arms was counted for 10 min. (B, D) Along with the 10-min measurement, the rate of entry into different arms was measured (mean ± SEM, n = 8 for p35f/f, CamkII-p35cKO and Dlx-p35cKO mice, ns, not significant, Mann–Whitney U-test).

## Data Availability

The details of sampling methods and the definition of variables are available from the corresponding author upon request.

## Abbreviations

Cdk5: Cyclin-dependent kinase 5
KO: Knockout
p35cKO: P35 conditional knockout
LTD: Long-term depression
LTP: Long-term potentiation
TBS: Theta burst stimulation
fEPSPs: Field excitatory postsynaptic potentials
I-O: Input–output
PPF: Paired-pulse facilitation
