# Supplementary figures and images for: Involvement of Cdk5 activating subunit p35 in synaptic plasticity in excitatory and inhibitory neurons
Source: Mol Brain. 2022 Apr 28;15:37. doi: 10.1186/s13041-022-00922-x (PMC9052517; doi:10.1186/s13041-022-00922-x)

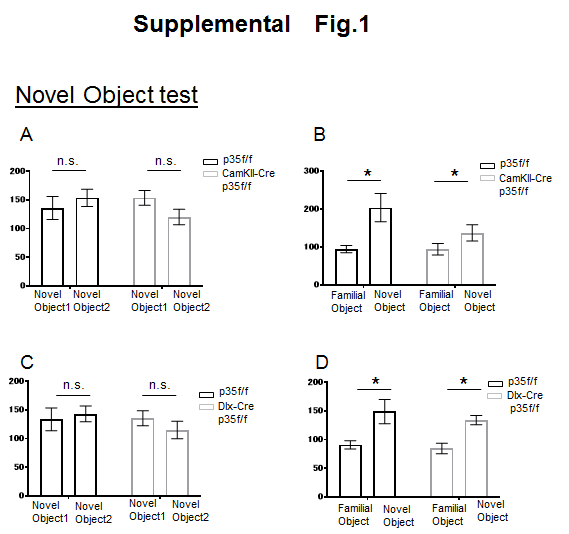

Supplement: Supplementary file 1 — Additional file 1: Figure S1. Novel object recognition (A, C) The mouse was placed in the open field in which the object was placed, and the time spent on the object was measured for 10 min. (B, D) After (A, C), a novel object was placed in the other corner and the time spent by the mouse on a familiar or novel object was measured for 10 min. (mean ± SEM, n = 8 for p35f/f, CamkII-p35cKO and Dlx- p35cKO mice,*p < 0.05 for object two-way repeated- measures ANOVA, ns, not significant). [file 13041_2022_922_MOESM1_ESM.tif]

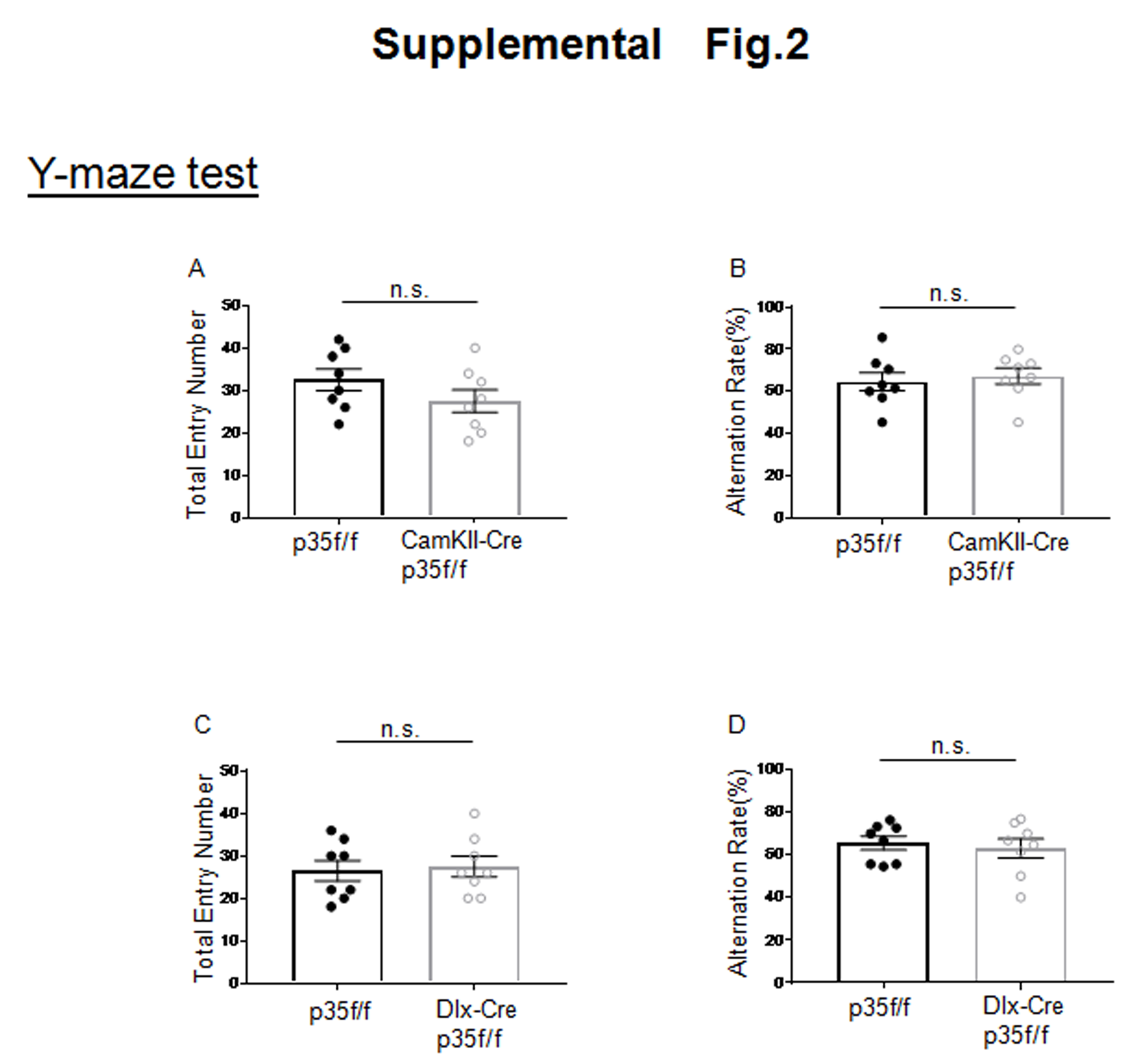

Supplement: Supplementary file 2 — Additional file 2: Figure S2. Y-maze test. (A, C) The number of times the mouse entered the three arms was counted for 10 min. (B, D) Along with the 10-min measurement, the rate of entry into different arms was measured (mean ± SEM, n = 8 for p35f/f, CamkII-p35cKO and Dlx-p35cKO mice, ns, not significant, Mann–Whitney U-test). [file 13041_2022_922_MOESM2_ESM.tif]
